# Low-grade oncocytic tumour (LOT) of the kidney is characterised by GATA3 positivity, FOXI1 negativity and mTOR pathway mutations

**DOI:** 10.3389/pore.2023.1610852

**Published:** 2023-02-01

**Authors:** Tongbing Chen, Yan Peng, Ting Lei, Chao Wu, Hui Wang, Yongqiang Shi

**Affiliations:** Department of Pathology, The Third Affiliated Hospital of Soochow University, Changzhou First People’s Hospital, Changzhou, China

**Keywords:** immunohistochemistry, clinicopathological characteristics, mTOR, differential diagnosis, low-grade oncocytic tumour (LOT) of the kidney

## Abstract

**Aims:** We present a 5-case series of low-grade oncocytic tumour of the kidney to further discuss their clinicopathological characteristics.

**Methods and results:** Five patients were included in this study. There were three females and two males aged 45–66 years, with a median age of 65 years. Four tumours were located in the right kidney, and one was located in the left kidney. Most of the tumour sections were yellow-brown in colour. Tumour sizes ranged from 2.5 to 4.5 cm, with a median size of 3 cm. Microscopically, the tumours were well-circumscribed but lacked a fibrous capsule; the tumours consisted of monomorphous oncocytic cells arranged mainly in solid and nested architectural patterns. The tumour cells had uniformly round to oval nuclei and often had perinuclear halos but lacked significant irregularities. Immunohistochemically, the tumour cells showed a diffuse and strong positivity for CK7 and were negative for CD117. The tumour cells were also positive for GATA3, E-cadherin, Pax-8, Succinate dehydrogenase B (SDHB) and Fumarate hydratase (FH), and negative for vimentin, Carbonic anhydrase 9 (CA9), CD10, P504s, CK20, TFE3, TFEB, HMB45, ALK and Forkhead box protein I1 (FOXI1). Next-generation sequencing identified genetic variations in these tumours, including *MTOR* gene mutations (4/5) and *PIK3CA* gene mutation (1/5). All patients were alive without disease progression at a median follow-up of 32 months (range 10–57 months).

**Conclusion:** LOT is an emerging renal entity of indolent behaviour that has morphologic overlap with some renal tumours with eosinophilic cytoplasm, primarily with oncocytoma and eosinophilic variant of chromophobe renal cell carcinoma. Familiarity with the distinctive morphological features, immunophenotype and molecular genetics of LOT helps avoid misdiagnosis.

## Introduction

Low-grade oncocytic tumour (LOT) of the kidney is an emerging renal entity that was first described by Trpkov K and Hes O in 2019 (1). This low-grade entity is composed of oncocytic tumour cells, and characterized by a CK7-positive/CD117-negative immunoprofile (2). Several case series have been reported. In the current study, we present five patients with LOT, and the clinical, histological, immunophenotypic and molecular features were analysed. The aim of our study was to further discuss the clinicopathological features and differential diagnosis of LOT.

## Materials and methods

### Case selection

From 2017 to 2021, 876 renal tumours were processed in The Third Affiliated Hospital of Soochow University/Changzhou First People’s Hospital. A series of 62 oncocytic renal tumours were identified, including 19 cases of eosinophilic variant of chromophobe renal cell carcinoma (E-ChRCC), 13 cases of renal oncocytoma, 16 cases of papillary renal cell carcinoma (PRCC) with eosinophilic features, 8 cases of clear cell renal cell carcinoma (CCRCC) with eosinophilic features, and 6 cases of unclassified oncocytic renal tumour. We only identified 5 tumours as LOT characterized by CK7 positivity and CD117 negativity. Of the five cases, four were originally diagnosed as E-ChRCC, and one as renal oncocytoma. The clinical and pathological findings were obtained from the medical records and pathology reports. Follow-up information was obtained by direct telephone communication with the patients and/or their relatives.

### Immunohistochemical analysis

Immunohistochemical staining was performed on 4-µm-thick formalin-fixed, paraffin-embedded (FFPE) tissue sections using a Roche Benchmark XT Automated Staining System. The antibodies used in this study (Supplementary Table S1) included CK7 (EP16, prediluted, ZSGB-BIO), CD117 (YR145, prediluted, MXB Biotechnologies), Carbonic anhydrase 9 (CA9) (H-11, prediluted, ZSGB-BIO), CD10 (MX002, prediluted, MXB Biotechnologies), P504s (13H4, prediluted, ZSGB-BIO), Pax-8 (EP298, prediluted, MXB Biotechnologies), vimentin (UMAB159, prediluted, ZSGB-BIO), E-cadherin (MX020, prediluted, MXB Biotechnologies), HMB45 (HMB45, prediluted, MXB Biotechnologies), CK20 (EP23, prediluted, ZSGB-BIO), Succinate dehydrogenase B(SDHB) (OTI1H6, prediluted, ZSGB-BIO), Fumarate hydratase(FH) (OTI1F10, prediluted, ZSGB-BIO), ALK (5A4, prediluted, MXB Biotechnologies), TFE3 (EP285, prediluted, ZSGB-BIO), TFEB (OTI2C1, 1:500, OriGene), GATA3 (EP368, prediluted, ZSGB-BIO), Forkhead box protein I1 (FOXI1) (EPR22940-151, 1:100, Abcam) and Ki-67 (UMAB107, prediluted, ZSGB-BIO). Positive and negative controls were used for each antibody. Immunoreactivity was scored by the percentage of positive tumour cells as follows: <1% (negative), 1%–50% (focal positive), and > 50% (diffusely positive).

### Next-generation sequencing

Next-generation sequencing (NGS) was performed by Illumina MiSeq (Illumina). The tissue DNA was extracted from FFPE tumour tissues using QIAamp DNA FFPE tissue kit (Qiagen, Hilden, Germany). Fragments between 200 and 400 bp from the sheared tissue DNA were purified (Agencourt AMPure XP Kit, Beckman Coulter, CA, United States), hybridized with capture probes baits, selected with magnetic beads, and amplified. Target capture was performed using a commercial panel consisting of 520 genes (Supplementary Table S2) chosen by Guangzhou Burning Rock Biotech Ltd. Sequence data were mapped to the reference human genome (hg19) using Burrows-Wheeler Aligner version 0.7.10. Local alignment optimization, duplication marking and variant calling were performed using Genome Analysis Tool Kit version 3.2, and VarScan version 2.4.3. Base calling in tissue samples required at least 8 supporting reads for single nucleotide variations (SNVs) and 2 and 5 supporting reads for insertion-deletion variations (Indels), respectively. Copy number variations (CNVs) were analyzed based on the depth of coverage data of capture intervals.

## Results

### Clinical features

The clinical features of the five patients are summarized in Table 1. There were three females and two males with ages ranging from 45 to 66 years (mean, 59 years; median, 65 years). All tumours were detected during a physical examination. Three patients underwent partial nephrectomy, and two patients underwent radical nephrectomy. Four tumours were located in the right kidney, and one was located in the left kidney. The tumour’s cut surface was mostly yellow‒brown in colour. Tumour size ranged from 2.5 to 4.5 cm in the maximal diameter (mean, 3.3 cm; median, 3 cm). No adjunctive treatment was administered. All patients had no evidence of local recurrence or distant metastasis during follow-up, which ranged from 10 to 57 months (mean, 34 months; median, 32 months).

**TABLE 1:** Clinicopathological features of low-grade oncocytic tumour (LOT) of the kidney.

Case	Age	Sex	Site	Size (mm)	Type of surgery	Status	Follow up (months)
1	45	Female	Right	25	Partial	Alive no evidence of disease	57
2	65	Male	Right	30	Partial	Alive no evidence of disease	53
3	66	Female	Right	35	Radical	Alive no evidence of disease	32
4	56	Male	Left	45	Partial	Alive no evidence of disease	21
5	65	Female	Right	30	Radical	Alive no evidence of disease	10

### Pathological features

**FIGURE 1:**
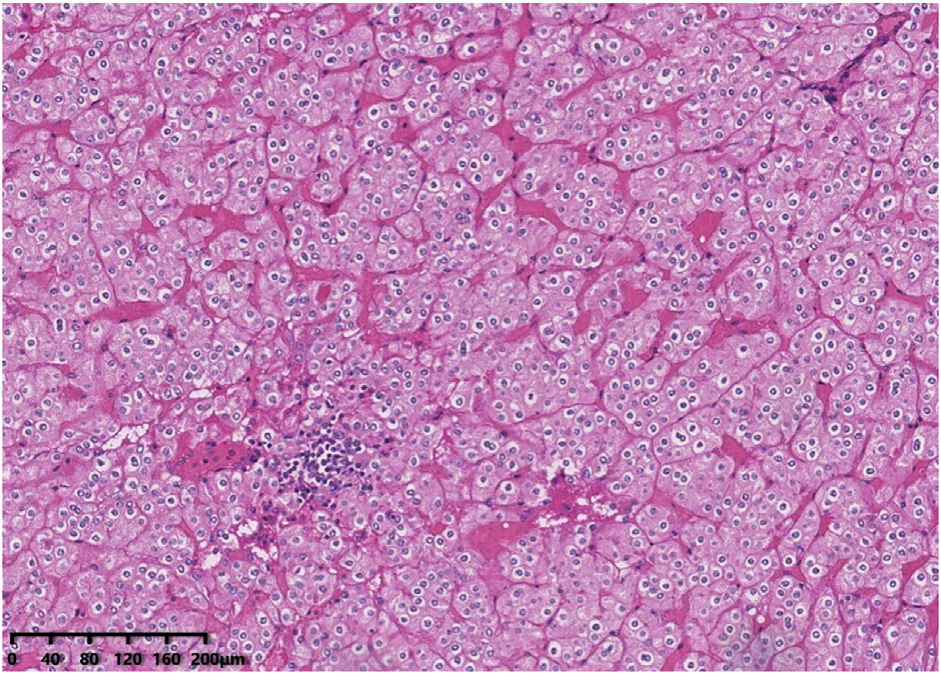
Histological features of low-grade oncocytic tumour (LOT) of the kidney. The tumour cells demonstrated a nested growth pattern. There are abundant thin-walled vessels around the tumour cell nest. Lymphocytic clusters were seen in this area. The tumour cells had perinuclear halos but lacked significant irregularities. The nucleoli were slightly conspicuous.

**FIGURE 2:**
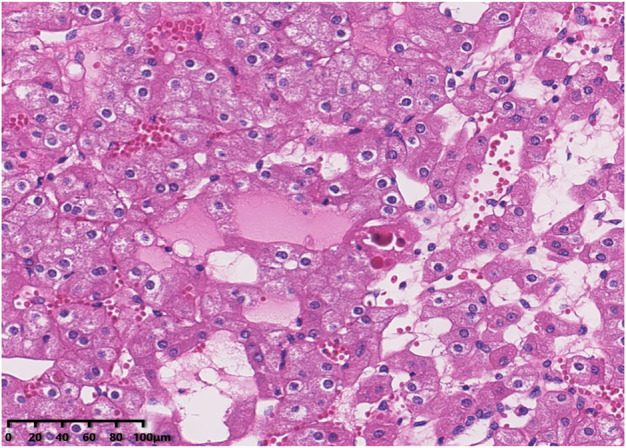
The tumour cells demonstrated a microcytic growth pattern.

**FIGURE 3:**
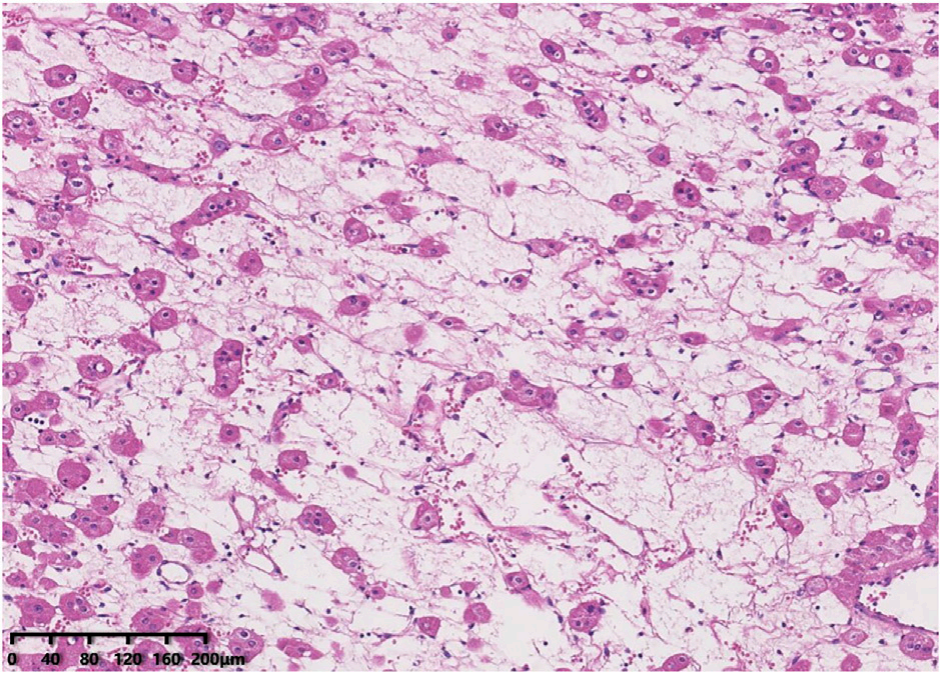
Oedematous stromal areas are presented, with scattered single-cell arrangement in these areas.

Histologically, all tumours were well circumscribed but lacked a fibrous capsule. The growth patterns included solid, nested (Figure 1), trabecular and microcystic growth (Figure 2). There were abundant thin-walled vessels around the tumour cell nest (Figure 1). Lymphocytic clusters (Figure 1) were observed in one tumour (Case 3). One tumour (Case 4) exhibited thick-walled vessels. Oedematous stromal areas were seen in two tumours (Case 2 and Case 4), with scattered single-cell arrangement in these areas (Figure 3). Focal fresh haemorrhage was also noted in three tumours (Cases 2, 3 and 4). No necrosis was observed in any of the tumours. The tumour cells had indistinct cell membranes, monomorphous oncocytic cytoplasm, and uniformly round to oval nuclei without mitotic activity. The tumour cells had perinuclear halos (Figure 1) but lacked significant irregularities. The nucleoli were slightly conspicuous (Figure 1).

**FIGURE 4:**
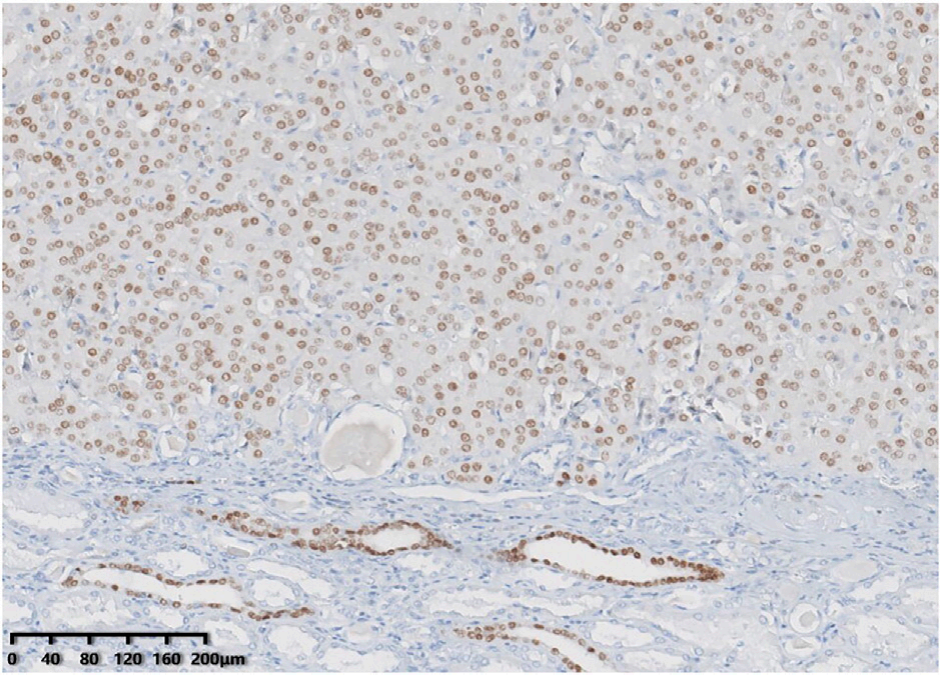
GATA3 showed consistent positivity in low-grade oncocytic tumour (LOT) of the kidney.

Immunohistochemically, all tumours showed diffuse and strong positivity for CK7 and were negative for CD117. GATA binding protein 3 (GATA3) (Figure 4), E-cadherin and Pax-8 were also present in all neoplasms. SDHB and FH were retained. All tumour cells were negative for vimentin, CA9, CD10, P504s, CK20, TFE3, TFEB, HMB45, ALK and FOXI1. The Ki-67 index was less than 3%. NGS identified genetic variations in these tumours (Table 2). Four tumours had a *MTOR* gene exon 53 p.L2427Q mutation [c.7280T>A (p.Leu2427Gln)], and one tumour had a *PIK3CA* gene exon 10 p.E542K mutation [c.1624G>A (p.Glu542Lys)].

**TABLE 2:** Genetic variations in the five adults with low-grade oncocytic tumour (LOT) of the kidney.

Case	Gene	Mutation_type	Description	Allele fraction (%)	Depth	Exon_number	Chromosome	Position	Hgvs_c	Hgvs_p
1	PRKDC	synonymous_variant	p.Y2483=	43.70	1389	55	8	48752579	c.7449C>T	p.Tyr2483=
	APC	stop_gained	p.R283*	17.26	2132	9	5	112151204	c.847C>T	p.Arg283*
	KDR	missense_variant	p.I519V	47.06	1719	12	4	55972089	c.1555A>G	p.Ile519Val
	EP300	missense_variant	p.P210S	45.35	3063	2	22	41513724	c.628C>T	p.Pro210Ser
	MTOR	missense_variant	p.L2427Q	20.92	2510	53	1	11174395	c.7280T>A	p.Leu2427Gln
	SPEN	missense_variant	p.D303V	47.95	1293	4	1	16235842	c.908A>T	p.Asp303Val
2	MTOR	missense_variant	p.L2427Q	9.34	1413	53	1	11174395	c.7280T>A	p.Leu2427Gln
3	MTOR	missense_variant	p.L2427Q	18.62	1847	53	1	11174395	c.7280T>A	p.Leu2427Gln
4	PIK3CA	missense_variant	p.E542K	24.85	1356	10	3	178936082	c.1624G>A	p.Glu542Lys
	CDK4	synonymous_variant	p.A16=	35.69	1303	2	12	58145453	c.48C>T	p.Ala16=
5	PDGFRB	synonymous_variant	p.V480=	44.71	2098	10	5	149509459	c.1440G>A	p.Val480=
	MTOR	missense_variant	p.L2427Q	17.73	1743	53	1	11174395	c.7280T>A	p.Leu2427Gln
	JAK3	splice_acceptor_variant	c.1787-2A>G	52.48	747	14	19	17946862	c.1787-2A>G	
	FAT1	intron_variant	c.13139-7C>T	43.38	989	27	4	187510381	c.13139-7C>T	

Abbreviations: Hgvs, human genome variation society.

## Discussion

Low-grade oncocytic tumour (LOT) of the kidney has emerged as a new diagnostic entity in renal tumour pathology in recent years (2–13). LOT is now enrolled in a new tumour subgroup called other oncocytic tumours of the kidney in the fifth edition of the WHO classification of urinary and male genital tumours (14), and is defined as a neoplasm with bland low-grade nuclei, diffuse strong CK7 labelling, and negative CD117 labelling. From 2017 to 2021, the incidence rates of LOT in our hospital were approximately 0.57% among renal tumours and 8% among oncocytic tumours in our study, while the incidence rates reported previously were 0.18% (4), 0.35% (5) and 0.17% (7) among renal cell tumours, respectively.

The clinicopathological features of LOT in this study are highly consistent with those of previously published cases. The tumours are found accidentally during physical examination in older patients as a single tumour, have a slight female predilection and have an indolent behaviour. Several LOTs have been reported in patients with tuberous sclerosis complex (TSC) (5, 6, 8) or end-stage renal disease (ESRD) (5). Multiple and/or bilateral renal tumours have been discovered (5, 6). Individual patients have died from unrelated diseases (5, 8, 12). The neoplasms in this study were typically well-circumscribed solid tumours consisting of monomorphous oncocytic cells arranged mainly in solid and nested architectural patterns, with no papillary growth pattern. The tumour cells were monomorphous with indistinct cell membranes and had an eosinophilic cytoplasm and prominent round to oval nuclei that lacked significant irregularities and often had perinuclear halos. Cystic change and central scarring are only seen in larger tumours (3) in general. Individual neoplasms have perirenal fat infiltration (3). Renal tubules can be entrapped at the periphery in individual tumours (4). Spindle elongated tumour cells are also observed, especially in hypocellular areas with oedematous stroma, such as cell culture growth. Muller–Mowry colloidal iron staining of LOT was either negative or only luminally positive (2, 3).

All the five tumours in this study had a typical immunophenotype characterized by diffuse positivity for CK7 and negativity for CD117, and were also positive for PAX-8, E-cadherin, FH and SDHB, but negative for vimentin, CD10, P504s, CA9, CK20, TFE3, TFEB, HMB45, and ALK. Rare neoplasms can show weak focal CD117 staining (2). Vimentin, CD10 and P504s can be focally positive (3, 4). BerEP4 (2, 4), MOC31 (2), CyclinD1 (4), 4EBP1 and S6K (8, 9) are also positive in LOT. Morini et al. (9) found no expression of FOXI1 in LOT. FOXI1 is a member of the forkhead transcription factor family, and high expression of FOXI1 has been found in restricted normal cell types, such as renal intercalated cells (ICs). Research has shown that FOXI1 is a potential biomarker of IC-related renal tumours, such as ChRCC and renal oncocytoma (15). We also identified negativity for FOXI1 in the current study. GATA3 belongs to the family of transcription factors that recognizes G-A-T-A nucleotide sequences in the target gene and is mostly used as a marker for breast and urothelial carcinomas. Studies (16) have found that GATA3 is expressed in distal nephrons, 51% of ChRCCs and 17% of oncocytomas. GATA3-positive reactions have also been documented in clear cell papillary renal cell tumour (17) and the recently recognized papillary renal neoplasm with reverse polarity (18). Therefore, GATA3 is not an entirely specific marker for one entity of renal cell neoplasms. Researches (12, 19) recently noted consistent GATA3 immunohistochemical positivity in LOT. We also observed GATA3 positivity in this study. Therefore, it can be inferred that the findings of FOXI1 negativity and consistent GATA3 positivity further expand the expected pattern of immunohistochemical markers in LOT.

Reports on the genetic analysis and molecular pathology of LOT are limited. Trpkov et al. (2) initially found that there were deletions at 19p13.3, 1p36.33 and 19q13.11 in some LOT patients by using array comparative genomic hybridization (ACGH). *CCND1* rearrangements were not found in LOT by using fluorescence *in situ* hybridization (FISH) (5). An increasing number of genetic tests have identified genetic mutations in LOT patients, including *RHEB* (8), *MTOR* (7–13), *TSC1* (6–9, 11–13), *TSC2* (11, 12), and *PIK3CA* (12), which are primarily involved in the *mTOR* pathway. The *PI3K/AKT/mTOR* signalling pathway plays a key role in cell survival and growth, *mTOR* is the master regulator of cell metabolism and growth, and acts through two different multiprotein complexes, namely, mTOR complex 1 (mTORC1) and mTOR complex 2 (mTORC2). mTORC1 is regulated by the tuberous sclerosis complex (TSC) (12). 4EBP1 and S6K expressed in LOT are the main downstream effectors of mTORC1 (20). All of these results confirm that LOT is a *mTOR* pathway mutation-associated renal tumour. We also found *MTOR* gene mutations in four tumours and an uncommon *PIK3CA* gene mutation in one tumour in this study.

Pivovarcikova et al. (21) discussed three TSC/mTOR pathway mutation-associated eosinophilic renal tumours, including eosinophilic solid and cystic renal cell carcinoma (ESC RCC), eosinophilic vacuolated tumours (EVT) and LOT. ESC RCC (22) is arranged in solid areas of eosinophilic cells with cytoplasmic stippling combined with cystic spaces lined by similar cells with a hobnail-shaped configuration. The tumour typically demonstrates the CK7-/CD117-/CK20+/vimentin+ immunophenotype. EVT demonstrates a solid growth architecture, large eosinophilic cells with distinct intracytoplasmic vacuoles, prominent cell membranes, large nuclei with prominent nucleoli, a CK7-/CD117+/CD10+/cathepsin K+ immunophenotype (23), and sporadic *TSC/MTOR* mutations (24). Recently, Xia et al. (13) further explored the molecular characteristics of *TSC/MTOR*-associated eosinophilic renal tumours, which included ESC RCC, EVT, LOT and unclassified renal tumours with *TSC/MTOR* mutations (*TSC-mt* RCC-NOS). They observed a specific trend in *TSC/MTOR* mutation in different tumours. ESC RCC and *TSC*-mt RCC-NOS displayed consistent *TSC1/TSC2* mutations, EVT demonstrated equal mutation distributions in *TSC* and *MTOR* genes, and all LOT cases but one bearing *TSC1* mutation displayed *MTOR* gene mutations.

LOT shows some similarities with oncocytoma and E-ChRCC. Oncocytoma often contains the CD117+/CK7− immunophenotype, and commonly exhibits recurrent chromosomal losses (1, 14, 21, X, Y) (14). E-ChRCC is characterized by eosinophilic tumour cells with raisinoid nuclei and perinuclear haloes and demonstrates CK7+/CD117+ immunophenotype. Some tumours are associated with Birt-Hogg-Dubé (BHD) syndrome harbouring *FLCN* germline mutations. Hybrid oncocytic/chromophobe tumour (HOCT) was initially described in patients with BHD syndrome harbouring *FLCN* gene mutations (14). The other differential entities include SDH-deficient RCC, TFEB translocation RCC, and oncocytic type of FH-deficient RCC. SDH-deficient RCC is negative for CK7, and loss of SDHB protein as determined by IHC (25). TFEB translocation RCC (26) is positive for cathepsin K, HMB45, Melan A, and TFEB. Molecular genetic analyses may detect *TFEB* gene expression. A new oncocytic type of FH-deficient RCC (27) exhibits variable cytoplasmic vacuolation, IHC shows strong nucleocytoplasmic 2SC positivity, PAX8 positivity and loss of FH expression, and can identify *FH* gene mutations. The broader differential types include epithelioid angiomyolipoma, CCRCC and PRCC with eosinophilic features, which are usually easily distinguished from LOT.

In summary, we present a 5-case series of LOT, an emerging entity of renal tumour with indolent clinical behaviour, which often presents a diagnostic challenge because renal tumours with oncocytic cytoplasm have a wide morphologic spectrum. The remarkable morphological features, immunohistochemical profile positive for CK7 and GATA3, and absent (or rarely weak) expression of CD117 and FOXI1 may help to avoid misdiagnosis. In challenging cases, the molecular genetic features of *TSC/mTOR* pathway mutations help to make the correct diagnosis.

## Data Availability

The datasets presented in this study can be found in online repositories. The names of the repository/repositories and accession number(s) can be found in the article/Supplementary Material.
