# Supplementary material for: Low-grade oncocytic tumour (LOT) of the kidney is characterised by GATA3 positivity, FOXI1 negativity and mTOR pathway mutations
Source: Pathol Oncol Res. 2023 Feb 1;29:1610852. doi: 10.3389/pore.2023.1610852 (PMC9928737; doi:10.3389/pore.2023.1610852)
Supplement: Supplementary file 1 [file Table1.DOCX]

**Table S1. Targeted Immunohistochemical antibodies**

| Antibody | Clone | Dilution | Company |
| --- | --- | --- | --- |
| CK7 | EP16 | prediluted | ZSGB-BIO |
| CD117 | YR145 | prediluted | MXB Biotechnologies |
| Carbonic anhydrase 9(CA9) | H-11 | prediluted | ZSGB-BIO |
| CD10 | MX002 | prediluted | MXB Biotechnologies |
| P504s | 13H4 | prediluted | ZSGB-BIO |
| Pax-8 | EP298 | prediluted | MXB Biotechnologies |
| vimentin | UMAB159 | prediluted | ZSGB-BIO |
| E-cadherin | MX020 | prediluted | MXB Biotechnologies |
| HMB45 | HMB45 | prediluted | MXB Biotechnologies |
| CK20 | EP23 | prediluted | ZSGB-BIO |
| SDHB | OTI1H6 | prediluted | ZSGB-BIO |
| FH | OTI1F10 | prediluted | ZSGB-BIO |
| ALK | 5A4 | prediluted | MXB Biotechnologies |
| TFE3 | EP285 | prediluted | ZSGB-BIO |
| TFEB | OTI2C1 | 1:500 | OriGene |
| GATA3 | EP368 | prediluted | ZSGB-BIO |
| FOXI1 | EPR22940-151 | 1:100 | Abcam |
| Ki-67 | UMAB107 | prediluted | ZSGB-BIO |
