# Supplementary material for: Low-grade oncocytic tumour (LOT) of the kidney is characterised by GATA3 positivity, FOXI1 negativity and mTOR pathway mutations
Source: Pathol Oncol Res. 2023 Feb 1;29:1610852. doi: 10.3389/pore.2023.1610852 (PMC9928737; doi:10.3389/pore.2023.1610852)
Supplement: Supplementary file 2 [file Table2.DOCX]

**Table S2. Targeted next-generation sequencing using a panel of 500 genes**

| *ABL1* | *ABL2* | *ABRAXAS1* | *ACVR1* | *ACVR1B* |
| --- | --- | --- | --- | --- |
| *AKT1* | *AKT2* | *AKT3* | **ALK* | *ALOX12B* |
| *AMER1* | *APC* | *AR* | *ARAF* | *ARFRP1* |
| *ARID1A* | *ARID1B* | *ARID2* | *ARID5B* | *ASXL1* |
| *ASXL2* | *ATM* | *ATR* | *ATRX* | *AURKA* |
| *AURKB* | *AXIN1* | *AXIN2* | *AXL* | *B2M* |
| *BAP1* | *BARD1* | *BBC3* | *BCL10* | *BCL2* |
| *BCL2L1* | *BCL2L11* | *BCL2L2* | *BCL6* | *BCOR* |
| *BCORL1* | *BIRC3* | *BLM* | *BMPR1A* | **BRAF* |
| *BRCA1* | *BRCA2* | *BRD4* | *BRD7* | *BRINP3* |
| *BRIP1* | *BTG1* | *BTG2* | *BTK* | *CALR* |
| *CARD11* | *CASP8* | *CBFB* | *CBL* | *CCND1* |
| *CCND2* | *CCND3* | *CCNE1* | **CD274* | **CD74* |
| *CD79A* | *CD79B* | *CDC73* | *CDH1* | *CDH18* |
| *CDK12* | *CDK4* | *CDK6* | *CDK8* | *CDKN1A* |
| *CDKN1B* | *CDKN1C* | *CDKN2A* | *CDKN2B* | *CDKN2C* |
| *CEBPA* | *CENPA* | *CHD1* | *CHD2* | *CHD4* |
| *CHEK1* | *CHEK2* | *CIC* | *CREBBP* | *CRKL* |
| *CRLF2* | *CSF1R* | *CSF3R* | *CSMD1* | *CSMD3* |
| *CTCF* | *CTLA4* | *CTNNA1* | *CTNNB1* | *CUL3* |
| *CUL4A* | *CXCR4* | *CYLD* | *CYP17A1* | *CYP2D6* |
| *DAXX* | *DCUN1D1* | *DDR1* | *DDR2* | *DICER1* |
| *DIS3* | *DNAJB1* | *DNMT1* | *DNMT3A* | *DNMT3B* |
| *DOT1L* | *DPYD* | *EED* | *EGFR* | *EIF1AX* |
| *EIF4E* | *EMSY* | *EP300* | *EPCAM* | *EPHA2* |
| *EPHA3* | *EPHA5* | *EPHA7* | *EPHB1* | *EPHB4* |
| *ERBB2* | *ERBB3* | *ERBB4* | *ERCC1* | *ERCC2* |
| *ERCC3* | *ERCC4* | *ERCC5* | *ERG* | *ERRFI1* |
| *ESR1* | **ETV4* | **ETV5* | **ETV6* | **EWSR1* |
| *EZH2* | **EZR* | *FANCA* | *FANCC* | *FANCD2* |
| *FANCE* | *FANCF* | *FANCG* | *FANCI* | *FANCL* |
| *FANCM* | *FAS* | *FAT1* | *FBXW7* | *FGF10* |
| *FGF12* | *FGF14* | *FGF19* | *FGF23* | *FGF3* |
| *FGF4* | *FGF6* | *FGF7* | **FGFR1* | **FGFR2* |
| **FGFR3* | *FGFR4* | *FH* | *FLCN* | *FLT1* |
| *FLT3* | *FLT4* | *FOXA1* | *FOXL2* | *FOXO1* |
| *FOXP1* | *FRS2* | *FUBP1* | *FYN* | *GABRA6* |
| *GATA1* | *GATA2* | *GATA3* | *GATA4* | *GATA6* |
| *GEN1* | *GID4* | *GLI1* | *GNA11* | *GNA13* |
| *GNAQ* | *GNAS* | *GPS2* | *GREM1* | *GRIN2A* |
| *GRM3* | *GSK3B* | *H3F3A* | *H3F3B* | *H3F3C* |
| *HDAC1* | *HDAC2* | *HGF* | *HIST1H1C* | *HIST1H2BD* |
| *HIST1H3A* | *H3C2* | *HIST1H3C* | *HIST1H3D* | *HIST1H3E* |
| *HIST1H3G* | *HIST1H3H* | *HIST1H3I* | *HIST1H3J* | *HIST2H3D* |
| *HIST3H3* | *HLA-A* | *HLA-B* | *HLA-C* | *HNF1A* |
| *HOXB13* | *HRAS* | *HSD3B1* | *HSP90AA1* | *ICOSLG* |
| *ID3* | *IDH1* | *IDH2* | *IFNGR1* | *IGF1* |
| *IGF1R* | *IGF2* | *IKBKE* | *IKZF1* | *IL10* |
| *IL7R* | *INHA* | *INHBA* | *INPP4A* | *INPP4B* |
| *INSR* | *IRF2* | *IRF4* | *IRS1* | *IRS2* |
| *JAK1* | *JAK2* | *JAK3* | *JUN* | *KAT6A* |
| *KDM5A* | *KDM5C* | *KDM6A* | *KDR* | *KEAP1* |
| *KEL* | *KIT* | *KLF4* | *KLHL6* | *KMT2A* |
| *KMT2C* | *KMT2D* | *KRAS* | *LATS1* | *LATS2* |
| *LMO1* | *LRP1B* | *LTK* | *LYN* | *MAF* |
| *MAGI2* | *MALT1* | *MAP2K1* | *MAP2K2* | *MAP2K4* |
| *MAP3K1* | *MAP3K13* | *MAPK1* | *MAPK3* | *MAX* |
| *MCL1* | *MDC1* | *MDM2* | *MDM4* | *MED12* |
| *MEF2B* | *MEN1* | *MERTK* | *MET* | *MGA* |
| *MIR21* | *MITF* | *MKNK1* | *MLH1* | *MLH3* |
| *MPL* | *MRE11* | *MSH2* | *MSH3* | *MSH6* |
| *MST1* | *MST1R* | *MTAP* | *MTOR* | *MUTYH* |
| *MYC* | *MYCL* | *MYCN* | *MYD88* | *MYOD1* |
| *NAV3* | *NBN* | *NCOA3* | *NCOR1* | *NCOR2* |
| *NEGR1* | *NF1* | *NF2* | *NFE2L2* | *NFKBIA* |
| *NKX2-1* | *NKX3-1* | *NOTCH1* | *NOTCH2* | *NOTCH3* |
| *NOTCH4* | *NPM1* | *NRAS* | **NRG1* | *NSD1* |
| *NSD2* | *NSD3* | *NT5C2* | *NTHL1* | **NTRK1* |
| **NTRK2* | **NTRK3* | *NUP93* | *NUTM1* | *P2RY8* |
| *PAK1* | *PAK3* | *PAK5* | *PALB2* | *PARP1* |
| *PARP2* | *PARP3* | *PAX5* | *PBRM1* | *PCDH11X* |
| *PDCD1* | *PDCD1LG2* | *PDGFRA* | *PDGFRB* | *PDK1* |
| *PGR* | *PHOX2B* | *PIK3C2B* | *PIK3C2G* | *PIK3C3* |
| *PIK3CA* | *PIK3CB* | *PIK3CD* | *PIK3CG* | *PIK3R1* |
| *PIK3R2* | *PIK3R3* | *PIM1* | *PLCG2* | *PLK2* |
| *PMS1* | *PMS2* | *PNRC1* | *POLD1* | *POLE* |
| *PPARG* | *PPM1D* | *PPP2R1A* | *PPP2R2A* | *PPP6C* |
| *PRDM1* | *PREX2* | *PRKAR1A* | *PRKCI* | *PRKDC* |
| *PRKN* | *PTCH1* | *PTEN* | *PTPN11* | *PTPRD* |
| *PTPRO* | *PTPRS* | *PTPRT* | *QKI* | *RAB35* |
| *RAC1* | *RAD21* | *RAD50* | *RAD51* | *RAD51B* |
| *RAD51C* | *RAD51D* | *RAD52* | *RAD54L* | **RAF1* |
| *RARA* | *RASA1* | *RB1* | *RBM10* | *RECQL4* |
| *REL* | **RET* | *RHEB* | *RHOA* | *RICTOR* |
| *RIT1* | *RNF43* | **ROS1* | *RPA1* | *RPS6KA4* |
| *RPS6KB2* | *RPTOR* | **RSPO2* | *RUNX1* | *RUNX1T1* |
| **SDC4* | *SDHA* | *SDHAF2* | *SDHB* | *SDHC* |
| *SDHD* | *SETD2* | *SF3B1* | *SGK1* | *SH2B3* |
| *SH2D1A* | *SHQ1* | **SLC34A2* | *SLIT2* | *SLX4* |
| *SMAD2* | *SMAD3* | *SMAD4* | *SMARCA4* | *SMARCB1* |
| *SMARCD1* | *SMO* | *SNCAIP* | *SOCS1* | *SOX10* |
| *SOX17* | *SOX2* | *SOX9* | *SPEN* | *SPOP* |
| *SPTA1* | *SRC* | *SRSF2* | *STAG2* | *STAT3* |
| *STAT4* | *STAT5A* | *STAT5B* | *STK11* | *STK40* |
| *SUFU* | *SYK* | *TAF1* | *TBX3* | *TCF3* |
| *TCF7L2* | *TEK* | *TENT5C* | *TERC* | *TERT* |
| *TET1* | *TET2* | *TGFBR1* | *TGFBR2* | *TIPARP* |
| *TMEM127* | **TMPRSS2* | *TNFAIP3* | *TNFRSF14* | *TOP1* |
| *TOP2A* | *TP53* | *TP63* | *TRAF2* | *TRAF7* |
| *TRIM58* | *TRPC5* | *TSC1* | *TSC2* | *TSHR* |
| *TYRO3* | *U2AF1* | *UGT1A1* | *VEGFA* | *VEGFB* |
| *VHL* | *WISP3* | *WRN* | *WT1* | *XIAP* |
| *XPO1* | *XRCC2* | *XRCC3* | *YAP1* | *YES1* |
| *ZBTB16* | *ZBTB2* | *ZNF217* | *ZNF703* | *ZNRF3* |

**Notes:** Single nucleotide variants (SNVs), insertion/deletion (INDEL) and copy number variation (CNVs) were analyzed. Gene fusion were also analyzed in genes marked *.
